# Guy's Hospital Reports. No. V.

**Published:** 1845-07

**Authors:** 


### GUY'S HOSPITAL REPORTS. 2nd Series, No. V. April, 1845. London, Highley.

THE papers in the present number are five, viz:—1, On the Pathology of Phthisis, by Dr. Addison—2, Cases in Medical Jurisprudence, by Mr. Alfred taylor—3, Illustrations of some of the Forms of Sudden Death from Disease, as it occurred in 19 individuals in the Manchester Workhouse, by Dr. Francis—4, Reports of Cases of Diseases of Children, treated in Guy's Hospital in 1843-4, by Dr. Golding Bird—and 5, Reports of Cases of Injuries of the Chest, to which no name is attached. We proceed to notice them *seriatim*; premising that this number fully sustains the high character of its predecessors for valuable information.

### I. ON THE PATHOLOGY OF PHTHISIS. By *T. Addison*, M.D. Senior Physician to the Hospital.

Believing that if ever we are to make any advances towards the discovery of the means of curing phthisis, it must be in the direction of an improved pathology, we regard investigations, such as Dr. Addison has long pursued in so indefatigable a manner, as of the highest importance. Advantageously do they contrast with the empirical and cruelly delusive proceedings so much in vogue at the present day! It is true that the farther we advance the more confirmed does the opinion seem to be, that there are forms of this disease for which a means of cure can never be devised; but it also becomes evident that, with these, others have been confounded, concerning which more favourable hopes may be entertained. Too little has been done, hitherto, in indicating the class of cases in which the suspension of the ravages, if not the cure of the disease, may be hoped for. That such cases not unfrequently occur is known to every practitioner, but any peculiarity in their nature has been insufficiently observed. In this point of view Dr. Addison's paper will be found very interesting; for, although we are scarcely prepared to admit the agency of inflammatory action to be so extensive as he states it to be, we are thankful for any contribution to the elucidation and more acurate determination of this long disputed and most important question. Dr. Evans, whose work we reviewed in our last number, attributed to it a general influence, but Dr. Addison insists upon its agency in certain forms of the disease only.

As a preliminary, he has long thought that the pathology of *pneumonia* itself should be more fully inquired into, and has, as our readers are aware, from time to time published the result of his researches upon this subject. Albuminous deposits, the consequences of pneumonia, are frequently found either in the form of small, detached, rounded masses, or diffused through the pulmonary tissue. They may remain quiescent and unchanged for an unlimited period; but if fresh inflammatory action arises around them, they, from their low degree of vitality, easily lose their cohesion and soften. The ease with which the simple form of pneumonia may be overlooked in *cachectic habits* explains why the nature of these deposits has been so frequently misunderstood. But even ordinary pneumonia, which has been duly recognised, may give rise to similar depositions without corresponding physical signs, which often arises from an accompanying pleuritic effusion masking these. Confusion again has arisen from the fact that such pneumonic induration is by far most frequently found in lungs which are likewise tuberculated. The probability of confounding this disease with disorganizing phthisis is much increased when it is situated, which however it is not commonly, at the apex, and when there is an excess of mucus in the dilated bronchial tubes. Three or four cases, in which the deposition of this grey, albuminous, matter gave rise to the symptoms of phthisis, have recently fallen under the author's notice, and are briefly adverted to. After which he goes on to observe:—

"From what has been advanced in this and former communications, I hope it may be permitted to arrange the several forms of disorganization of the lungs resulting from mere inflammation and its consequences under the head of PNEUMONIC PHTHISIS. This may be *acute:* the deposits and inflamed tissues softening down and disorganizing at once, without any attempt whatever being made at induration or repair; thereby constituting one form of acute or galloping consumption. It may be *acuto-chronic;* of which I would distinguish three varieties. 1. The inflammation, though more or less acute, is slower and more insidious in its course, and manifests some attempts at repair, as indicated by various stages and degrees of induration. The induration, nevertheless, is not complete; the pulmonary tissue continues to be friable; and, sooner or later, that is to say in a few weeks or months, softens down, and gives rise to excavation; most frequently by a somewhat slow ulcerative process; more rarely by an actual slough of greater or less portions of the indurated but still friable pulmonary tissue. 2. Inflammation may supervene upon or around ancient induration, leading to disorganization, either of the newly-inflamed tissue, of the old induration itself, or of both at the same time. Lastly, pneumonic phthisis may be *chronic;* of which I would also distinguish two varieties; 1st, that in which old indurations undergo a slow process of disintegration, giving rise to vomicæ; and 2nd, that very rare form of the disease, in which an insidious inflammation proceeds very slowly to convert a considerable portion of pulmonary tissue into grey induration, without any necessary excavation whatever."

*Tuberculo-Pneumonic Phthisis.*—" By this is meant a common form of the complaint, in which, although tubercles are present, the really efficient cause of the phthisical mischief is pulmonic inflammation. The tubercles sufficiently indicate the strumous or cachectic habit of the individual, and manifestly predispose to the inflammatory change; nevertheless they do not, beyond this, seem to be either primarily or essentially concerned in the serious changes observed to take place in the pulmonary tissue." Simple pulmonary *tubercle,* when of a grey colour, and vitreous semi-transparent appearance, and of a moderately hard and resisting consistence, is termed by the author *sthenic,* being much less prone to disintegration than the *asthenic* variety, which is of an opaque white, or yellowish colour, and of a softer and more friable consistency. It is the former variety usually found in cases of tuberculo-pneumonic phthisis. The so-called enlargement of tubercle results from an aggregation of simple tubercles, or the surrounding of simple tubercle by the products of inflammation. The former may be termed *compound tubercles,* which are much more prone to disintegration than the simple. When tubercles are numerous, the neighbouring air-cells frequently take on compensatory or excessive action, and such inequality of respiration is the only sign to be observed at an early period of the disease. The presence of tubercles also induces a tendency to congestion and inflammation of the lungs, especially in their vicinity; but, unless such tendency become aggravated by various causes, the tubercles seem to cause little or no inconvenience. This inflammation (whether occurring in the pulmonary tissue or in the mucous membrane of the air-passages), which is the ordinary commencement of this form of phthisis, is of a low and insidious kind, such as is observed in cachectic and scrofulous habits; and, as it progresses, the various physical signs, as feebleness or absence of murmur, bronchophony, tubular respiration, and dulness on percussion, arise more from its presence than from the extension of the tubercles, for they may all occur when these latter are absent. The pneumonic heat of skin, when present, is often remarkably correspondent to the rapidity and extent of the local changes ascertained by auscultation.

" The inflammatory changes which take place in tuberculated lungs resemble those resulting from inflammation of the lungs without tubercles. Although the red hepatization occurring in tuberculo-pneumonic phthisis very often passes quickly into softening, and the consequent formation of a cavity; nevertheless, when actual albuminous matter is thrown out, it, like that resulting from pulmonic inflammation without tubercle, usually manifests some attempts at repair; as indicated by more or less hardening and contraction of the deposit itself, and of the pulmonary tissue, into which it is effused. These results of inflammation have been very commonly, but erroneously, regarded as mere varieties of tubercular infiltration. * * * * * * * * * The attempts at repair, or, induration of the pneumonic deposits occurring in tuberculo-pneumonic phthisis, are commonly very imperfect, and not durable; so that the deposits, for the most part, sooner or later undergo a second change, by which they soften down and produce excavation. This softening, however, may take place days, weeks, months, or, I believe, even years after the original deposition. When the disease has proceeded to excavation, the natural cure of the ulcer thus produced, consists in the formation of a more or less dense and permanent lining membrane—the true cicatrix of such ulcers. Although this membrane may perhaps now and then remain passive and harmless for years; it most commonly happens, that the cicatrization is imperfect and incomplete; the efforts at repair fail; the albuminous material, which ought to form the membrane, softens down, and with it successive portions of the pulmonary tissue furnishing it; and thus the ulceration proceeds, till, exhausted by unceasing irritation and imperfect nutrition, the patient dies."

In some instances, no attempt is made at repair in the pneumonic deposits, which, as well as the surrounding tissue, soften down at once, producing one form of, what is usually a result of the asthenic variety of the disease, galloping consumption. On the other hand, diffuse inflammation attacking a lung studded with tubercles, may cause death before any albuminous deposits are made. This may be called *suffocative pneumonia*, being characterized by disproportionate dyspnœa and lividity of countenance, and often by rapid dissolution.

Our remedial means are purely preventive, and are such as improve and strengthen the general constitution, and secure the removal of all causes tending to excite inflammatory action in organs so critically situated. Where this has however arisen, it must be met by moderate depletion and active and continued counter-irritation. Bland diet, warm clothing, regulated temperature, or a milder and less variable climate than our own are required. When the inflammation is considerable and obstinate, mercury may be added to the above means: but affecting the constitution by this medicine has served in some cases to hasten ulterior disorganization. We are surprized that the author makes no use of antimony in this form of pneumonia, assuredly as useful and a far safer drug than mercury.

"The disease, by proper treatment and management, may often be arrested for a time; and not very unfrequently for years; the pneumonic changes already produced, remaining in a passive state, as determined by auscultation and percussion. It is but just, and of some importance, to admit; that although the general tendency to repair or induration in pneumonic deposits; and the absence of it in tubercle; are strongly marked, and sufficiently distinguishable; it is sometimes difficult or impossible to pronounce with certainty, whether the change in the pulmonic tissue, be the result of tubercles, or of a by-gone inflammation; neither is it at all times easy to decide whether the inflammatory changes, when sufficiently evident, have or have not been preceded by tubercle."

*Tubercular Phthisis.*—In the form of the disease just described the tubercles being of the *sthenic* kind, and little *compounded*, usually remain quiescent until disturbed by surrounding inflammation, which proves the principal source of destruction, first of the pulmonary tissue and consecutively of the tubercles. If the sthenic tubercles are much compounded, however, they may undergo primary disintegration, independently of the usual precursory pneumonia. The line of demarcation between such cases and tubercular phthisis, consisting of a preponderance of *asthenic* and compounded tubercles, is by no means precise. Dr. Addison ably describes the condition of these latter bodies prior to, and during the process of, softening. He inquires, also, to what is the softening due, and seems disposed to refer it to some process analogous to inflammation going on in the pulmonary tissue which is the immediate site of the deposit. Whether, however, it arises from this or a mere mechanical cause, it is certain it occurs in masses of, or even in single, asthenic tubercles, independently of any sign of inflammatory action in the *surrounding pulmonary tissue*. Still, although inflammatory action is not the immediate cause of softening in this form of tubercle, it is, when the disorganization is extensive, an almost invariable complication. It may extend from the vicinity of the tubercles to one or more inches, or it may arise at a distance from the tubercles, and is often accompanied by an inflammation of the pleura situated immediately over it. This inflammation is of an asthenic character, the albuminous matter being effused in the form of concrete or fluid pus, and no attempt at the reparation of ulceration, as sometimes occurs in the sthenic form, is made.

"The asthenic character, equally apparent in the tubercles and in the accompanying inflammation: whilst it establishes the influence of constitutional peculiarity, goes far to deprive us of all reasonable hope of ever being able successfully to combat this variety of phthisis, when once developed; for, without repudiating those rare exceptions to be met with in the history of the most fatal disorders; pathology alone, would lead me, however reluctantly, to subscribe to the opinion of those, who pronounce this form of phthisis at least, to be at once incurable and hopeless. *Prevention* I believe to be the golden rule in every form of phthisis; and especially in the tuberculo-pneumonic and tubercular varieties; but I think I may venture to predict, that if a remedy, either of prevention or of cure, be in store, as a mercy and blessing for future generations; that remedy will prove to be very different indeed, from the enervating expedients so indiscriminately employed at the present day."

Dr. Addison points out some appearances which have been very often mistaken for tubercles. Thus, in a lung which has long been the seat of irritation, the parietes of the bronchial tubes become so indurated and thickened, that their canals, on cutting across the minute ones, cease to be visible, and the truncated extremities have been mistaken for tubercles. So, too, the dilated portions of bronchial tubes, passing through an indurated lung, have been frequently mistaken for cavities containing pus, or for tubercles softening in the centre. An albuminous deposit in some of the smaller bronchial tubes, analogous to what is observed in croup, has been mistaken for tubercle. But the most frequent source of error is found in the bronchial tubes traversing the hepatized portion of a phthisical lung. Their parietes may be thickened, softened and dilated; and when they contain puriform mucus they are continually mistaken for tubercle.

Dr. Addison concludes his very interesting paper in these words:

"Should this very imperfect communication have the effect of awakening in the breasts of practitioners and students, a desire, more zealously and carefully to investigate the pathology of phthisis; its principal object will have been accomplished; whether such investigation tend to confirm or to refute the proposition, that, *inflammation constitutes the great instrument of destruction in every form of phthisis*."

Appended to this memoir, there are five plates of admirably-coloured lithographed drawings of diseased lungs, in various stages of inflammatory and tubercular change, and of the appearances liable to be mistaken for tubercular cavities. We have never seen more faithful representations of morbid structure.

### II. Cases in Medical Jurisprudence, with Remarks. By *Alfred S. Taylor.*

Whatever is calculated to improve our knowledge of the medical history of Poisons—unfortunately a too common instrument of murder in the present day—demands the studious attention of every one engaged in active practice. The following cases and observations will amply repay perusal, and serve to illustrate, in some very material points, the description which we gave of Prussic Acid in our last number, p. 481.

A commercial traveller was found dead in his bed at an inn. The body was lying in the recumbent position as in natural rest, and exhibited no traces of previous convulsive suffering. The bed-clothes were smoothly drawn up to the deceased's shoulders. There had been no vomiting; but the bowels had acted involuntarily, no doubt during the act of death, or perhaps immediately after it—a not unfrequent occurrence in animals that have been poisoned by Prussic acid. The eyes were open and particularly *bright.* About the mouth, there was a decided smell of prussic acid to be perceived. On a chair, at the back of the bed, but close to it, was a phial *with the cork in it.* It contained a small portion of a liquid that smelt strongly both of prussic acid and the essential oil of lemons.

*Dissection.*—We notice only the most striking phenomena. The fluid in the ventricles of the brain were strongly impregnated with the odour of the acid: equally so was the blood in the cavities of the heart. In the stomach also was found about half-a-pint of a viscid liquid, having a strong odour of the poison. The stomach and its contents were not forwarded to Mr. Taylor for examination, until the 12th day after the decease. They (the contents) were then turbid, of a brown colour, and had an extremely offensive odour—but certainly not of prussic acid. They were very cautiously distilled; but still no trace of the peculiar smell of this poison was perceptible; neither was its presence indicated by the appropriate tests—nitrate of silver and the green sulphate of iron with caustic potash.

Notwithstanding the absence of direct chemical evidence, there was every reason to believe that the death, in this case, was the result of Prussic acid, taken by the patient himself; and accordingly a verdict of *felo-de-se* was returned. It does not appear that the poison was detected either in the phial, or in the contents of the stomach, previously to the inquest being held; and, as we have already said, so long a time had elapsed before they were sent to Mr. Taylor, that all search for it then was fruitless. Prussic acid has been found in the stomach so late as *seven days* after death, and its odour has been perceived much longer; but, in this instance, *twelve* had elapsed. Here we may notice with advantage one or two important points connected with the history of this most destructive poison.

The peculiar odour may be wanting in a fluid, and yet the acid may be present in it, and discoverable by the proper tests: it is most necessary to remember this. On the other hand, the odour may be strong, and yet its presence may not be capable of detection by any known re-agent. Mr. Taylor remarks:

"The blood may have the odour of prussic acid, but none of the poison can be separated from it by distillation. I have remarked that the watery solution of the distilled product of the oil of bitter almonds with lime, after having been entirely precipitated by nitrate of silver, and filtered, retains the odour, commonly regarded as characteristic of prussic acid, as strongly as before. This shews that the odour, or an analogous odour, may exist where no test will act: in fact, the poison may be in too small a quantity for detection, since there is undoubtedly a limit to the operation of the tests." 43.

On the whole, it must be quite obvious to the candid and cautious jurisconsult that no *condemning* or *acquitting* importance ought to be attached to the mere presence or absence of the peculiar smell, apart from other evidence, in any case that warrants suspicion. In the recent case of Tawell, where the dissection of his victim took place only 18 hours after death, there was a striking discrepancy of opinion as to the *fact*. Three of the medical men present could not perceive the odour in the contents of the stomach, while two declared that they did perceive it! We need scarcely say that the mere presence of the smell, however distinct, in the stomach or in the blood, should never, *per se*, be admitted as perfectly conclusive evidence—however strong may be the presumption—of the existence of the poison, at least in criminal cases.

In has been conjectured, if not positively asserted, by Orfila and others, that prussic acid may be spontaneously generated by a re-action in the elements of organic fluids, in the act of distillation. Mr. Taylor however refuses to yield his assent to this opinion; nor does he think that a single instance can be quoted, where the production of this acid has taken place from the contents of the stomach of a person who had died from natural causes.

The circumstance that the peculiar smell of prussic acid may be very materially disguised by the admixture of certain essential oils—as those of turpentine, lemons, lavender, peppermint, &c.—should be remembered by medical men, as it may serve to put them on their guard not to be misled by the mere absence of the expected odour in suspicious cases. London porter also serves to conceal the odour of the poison more effectually than most fluids in ordinary use. Tawell used it for this end, and we have seen how well it partly served his purpose.

In a case of poisoning by prussic acid, recently reported by Mr. Crisp of Walworth, the *post-mortem* examination was not made before 70 hours after death; and then no odour could be detected in any part of the body. The most remarkable necroscopic feature in this case was the *violet colour* of the skin and abdominal and thoracic viscera, owing to the purple and blueish appearance of the blood—a phenomenon which has been observed in other similar instances.

The persistence of the peculiar smell seems to vary a good deal in different cases. It would seem that it disappears more rapidly in the human subject than in animals; and in them, although the odour may be present, the acid cannot always be obtained by distillation. Dr. Lonsdale found, in his experiments on animals, that it might be perceived for *eight* or *nine* days after death, although he could not detect its presence by chemical tests for more than *four;* and in the case of one of the seven epileptic patients, accidentally poisoned some years ago, in a Parisian hospital, Devergie states that the odour was perceived "huit jours apres l'ouverture du corps."

One of the most important questions, connected with the history of poisoning by prussic acid, is that respecting the length of time during which the patient may retain his consciousness, after having taken the fatal dose. It is of great consequence in a medico-legal point of view that physicians should be rightly informed on this subject. In the first case which we have related, it will be perceived that the deceased was not rendered insensible immediately he had swallowed (a large dose probably of) the poison; but that, after taking it from the phial—for no other vessel was found near—he had corked it and placed it on a chair at the back of the bed, and had then drawn the bedclothes smoothly up to his shoulders, and composed himself into a position of rest, inclined over to the left side. It will be remembered, too, that no appearance of the corpse indicated the occurrence of convulsions before death.* Mr. Taylor remarks that, judging from the results of experiments on animals, the extinction of life is (occasionally, at least) by no means instantaneous, even when the dose of the poison has been very large; and indeed that the rapidity of its destructive operation is certainly not always proportionate to the quantity that has been administered. On this point the following observations deserve attentive notice:

"Prussic acid, given in equal doses to animals, begins to operate with very unequal rapidity. In some instances I have observed that the effects were immediate: there was no appreciable interval, either to the person who held the animal or to myself, between the time of applying the poison to the tongue and the commencement of the symptoms. There were convulsions with opisthotonos in cats, and emprosthotonos in mice; but in several instances, although the animal appeared of equal strength, and the dose was the same, the symptoms did not commence until after the lapse of from fifteen to thirty seconds. The same may hold with respect to the human subject. There are few cases, however, in which the person has been seen to swallow the poison; and when this has accidentally happened, those present were incompetent to watch the effects. If, therefore, we are justified in applying the results of experiments on animals to the supposed action of the poison on the human subject, we must apply them as a whole, and not make a selection. There is great variety in the effects produced by other poisons on the human body, where opportunity for observation occurs, both as to the nature of the symptoms and the time at which they commence. This is seen in cases of poisoning by opium; and why may not the same irregularities occur with respect to prussic acid? In reference to this poison, the admission of only a few seconds longer for its operation in one case than in another would account for those results which have been frequently regarded as anomalous, and justifying a suspicion of murderous interference." 50.

We may therefore receive it as a demonstrated fact that we cannot predict, from the largeness of the dose in a given case, either in the human subject or in the lower animals, the exact period at which it will prove fatal; and we are further warranted in believing that a certain period—

---

* "In observing whether or not the hands be clenched, it must be remembered, that in cases of natural death, where the body has not been disturbed, the thumb is almost constantly found bent towards the base of the little finger in the palm of the hand, and covered by the fingers which are semiflexed over it. So uniformly is this appearance met with, that it was some years since proposed as a sign of death by MM. Villermé and Breschet." 56.

sufficient for the performance of some voluntary act or acts—may elapse between the swallowing of a large dose and the extinction of life.

Here we must allude to another point in the history of this poison, of no inconsiderable importance. It has been believed and asserted by many that convulsions invariably precede death from Prussic acid, and that some traces of these are almost always discoverable on the body of the deceased. This seems, indeed, to be nearly true as respects the lower animals, but certainly it does not hold uniformly in the case of human beings; for, on more occasions than one, the poor victim has been found in a most composed and sleep-like attitude, without any sign whatsoever of struggling or convulsion having taken place; and this too when there was every reason to believe that the act had been suicidal.

Let us return for a moment to Mr. Crisp's case, and we shall find in its report a confirmation of the statement now made.

"The body of the deceased (aged 42) was found lying on the right side of the bed, inclining to the left, the face suffused; but it does not appear that there was any thing remarkable in the attitude, or any thing indicative of convulsion or struggling; for such a condition would hardly have escaped Mr. Crisp's observation, and would have been noticed in the report. In the chamber-vessel, which had been pushed some distance under the right side of the bed, there was found a tumbler, with a two-ounce empty stoppered bottle; the stopper out of the bottle, and the printed label, with 'Prussic Acid' on it, floating in the urine. The quantity of poison taken could not be ascertained; but there was strong reason to believe that the dose was large. A question arose at the inquest, whether, after taking the fatal dose, the deceased could have put the tumbler into the chamber-vessel, have pushed this under the bed, and then turned himself over on the left side. There was no doubt from the evidence that this was a case of suicide, and that these acts had been performed by the deceased. It is highly probable, from the size of the bottle and other circumstances, that the dose of poison was much larger in Mr. Crisp's case, than in that here reported; yet it is to be observed there were no signs of convulsions in either instance; the acts would have occupied as much time in the one case as in the other; and hence it is fair to infer, that in neither could the loss of consciousness and voluntary power have been *immediate*." 53.

We cannot therefore doubt that a person is capable of voluntary exertion, to a certain amount, for a short time after he has swallowed a large dose of this most deleterious poison. But if a medical man were asked whether a person could perpetrate suicide by other means, after having taken Prussic acid, his answer would assuredly be in the negative. The following case, which is in this respect of some interest, occurred in London in April 1839. A young man was found hanging quite dead; and he had evidently taken prussic acid, for a cup was lying near him which had contained that poison. The medical practitioner, who gave evidence at the inquest, very properly inferred that the man did not swallow the poison until after he had adjusted the rope round his neck. It can hardly be admitted that a man should have power to adjust a rope and hang himself, after having taken a large dose of prussic acid; but undoubtedly a person might be found drowned with prussic acid in his stomach, and without the fact being incompatible with suicide. It is, however, a matter of doubt, determinable only by the special circumstances of the case, whether a man could destroy himself by fire-arms, after having taken this poison.

There is still one point more in the history of poisoning from prussic acid that calls for some notice. In the recent case of Delaney, tried for the murder of his wife, it was stated that the insensibility produced by the poison is, in almost every case, immediately preceded by a shriek, or scream. We would caution our readers not to attach too much importance to this assertion; for, in numerous experiments on animals, it has been found that death takes place without their uttering any cry whatever; and the same has been remarked in the case of the human subject, when the suicidal act has been committed in the presence of another person. On more than one occasion, the deceased has been known to utter a few words, rational in every respect and indeed declaratory of his dreadful deed, and then to fall back and die immediately afterwards. In one case, the party exclaimed to a person in his room, "It's gone;" and, in answer to a question put, said "I have taken it." He was again about to speak, but his articulation failed him; he became insensible, and expired. In another, an apothecary's assistant was the unhappy victim. He had gone to a cellar to procure a drug. A few minutes afterwards, he was heard to cry out "Hartshorn." Some persons hastened to the spot, and found him on the lower steps of the cellar, just uttering the words "Prussic acid:" life was extinct immediately afterwards. From these facts, we perceive that the assertion of Delaney, that his wife had spoken to him a few words after he heard her scream, *may have been* really true.

When the dose taken is only barely sufficient to destroy life, the fatal effect may not be produced for a considerable time. In the case of the Parisian epileptic patients, already referred to, each took a quantity equivalent to about 0·7 of the pure anhydrous acid, and they all died in from 30 to 45 minutes.

Mr. Taylor has appended some remarks on the *tests for prussic acid*, that deserve the notice of the medical chemist. For these, we must refer to the memoir itself.

### III. Illustrations of some of the Forms of Sudden Death. By *D. J. T. Francis*, M.B.

We invite the attention of all our readers to the following summary of Dr. Francis' very instructive communication, and beg of them to bear in mind the remarks which we have made on the treatment of Apoplexy in a previous page. They will find, in the present article, fresh proofs of the necessity of caution in the use of blood-letting in the medical management of a sudden *fit*, whether it be of an apoplectic nature or not.

Within a period of two years and five months, 1000 deaths occurred among the inmates of the Manchester workhouse, in which Dr. Francis is the resident medical officer. Of this number, nineteen were instances of what is usually understood by the term "sudden death;" which it is intended, with one or two exceptions, to apply, in the following narration, to those cases wherein life has become extinct within a quarter of an hour from the commencement of the fatal seizure. We proceed to analyse them; this it is the more easy to do, as they are arranged under separate heads.

I. *Cases in which the Cause of Death was seated in the Brain.*

Three examples are given. The first occurred in a man, æt. 56, who, although he had been disabled for four or five years by a partial palsy of the left arm and leg, continued in good general health. One day, while standing up in the middle of the yard, passing his urine (he had considerable difficulty in micturition), he was observed to glide easily to the ground: he gave two or three short cries, but there was no struggling. "Within two minutes after he fell, being close at hand, I saw him; but from this time there was no attempt at inspiration, the whole apparatus being quite motionless, although the heart continued to beat, pretty forcibly at first, and afterwards with a series of three or four faint pulsations, and gradually lengthening intervals, for nearly three minutes after I first saw him. The face was not remarkably livid: the pupils were somewhat, but not much, dilated. From these circumstances, an effusion of blood interfering with the respiratory portion of the medulla oblongata, and annihilating the perception of a 'besoin de respirer,' was predicted."

*Dissection.*—Extensive effusion of blood at the base of the Brain and around the medulla oblongata. Lateral ventricles empty; a few clots in the fourth ventricle. The arteries at the base were studded with opaque atheromatous spots; on gently throwing a current of water into the basilar artery, two jets issued from apertures in the posterior cerebral arteries. *Heart* sound, or nearly so. Some traces of an old pleuritic and pneumonic attack in the left lung. Abdominal viscera sound.

*Case* 2.—A man, æt. 58, having been absent for a minute in the water-closet, was heard to fall on the ground. He was found comatose; the breathing was slow and heavy; the countenance was of a sub-livid hue; the pulsations at the heart and wrist were full and powerful, and continued most distinctly for some time after all attempts at respiration had ceased. The pupils were at first contracted; and afterwards somewhat, but not greatly, dilated. Death took place in about eight minutes. This man had had an attack of partial hemiplegia six years before.

*Dissection.*—The lateral ventricles of the *Brain* contained a large quantity of bloody serum and florid clot. Great softening of the *fornix* and of the ventricular parietes. There was extensive effusion of recent coagulated blood at the base of the brain. The fourth ventricle was filled with a coagulum, which, after firmly investing the medulla oblongata, passed down the spinal canal, as far as it could be seen. There was no appearance of disease in the posterior cerebral or basilar arteries; but, when water was thrown into the latter, it escaped freely from the vessels in the fissure of Silvius, which were becoming opaque and hardened. *Lungs* almost entirely healthy. Pericardium adherent in nearly its whole extent. Left ventricle of the *heart* much thickened; valves sound. Both kidneys granular.

*Case* 3.—A man, æt. 54, a tailor, soon after eating a hearty breakfast of porridge and milk, and while sitting in the usual cross-legged posture at his work, suddenly complained of pain in the head, and fell from his seat, becoming almost immediately quite insensible. He had returned from the water-closet only a few minutes previously. He died within five minutes from the moment of the seizure.

*Dissection.*—All the sinuses of the *brain* were enormously distended: its substance throughout seemed healthy. Ventricles empty. No appearance of extravasation anywhere. After the brain was removed, several ounces of blood flowed from the spinal canal and the base. Both *lungs* were excessively gorged with fluid blood. The *heart* was small, pale, and flabby; left ventricle thin and lacerable; when cut into, there escaped about four ounces of dark fluid blood, much of which came from the aorta. The right side was not quite so full of blood; the ventricle containing a loose fibrinous clot, the only one found in the body. The abdominal viscera were dark and congested, but seemingly healthy otherwise.

*General Remarks.*—With respect to these three, and suchlike, cases, we may presume that the cause of death is pressure upon the *medulla oblongata*—whether applied immediately to the part from extravasation, or indirectly from the general distension of the cerebral mass with blood—and the consequent suspension of the function of Respiration. The following illustrative observations will be read with interest.

"Independently of the pathological interest which attaches to these cases, they were alike characterized by a remarkable symptom, which, as it was carefully observed, could not fail to arrest the attention;—I mean, the decided manner in which the action of the heart and the pulse at the wrist were prolonged, after the respiration had completely ceased. This circumstance was obviously due for its cause to the especial manner in which the hæmorrhage that had taken place had compressed the medulla oblongata, and so annihilated the function of the part which presides over the respiratory movements; whilst the heart, being less completely under the influence of that portion of the nervous system, had continued in its action, ceasing only from the obstructed circulation through the lungs. Experiment and previous pathological observation had long since established the fact of the dependence of the respiration upon integrity of the medulla oblongata; but the availability of the priority in cessation of the action of the heart or lungs, as a means of elucidating the causes of sudden death, appeared further to be an object well worthy of careful investigation." 97.

II. *Cases in which there was Congestion of the Right Heart and Lungs.*

*Case* 4.—A woman, æt. 73, was sitting upon the close-stool, to which she had walked unassisted, and suddenly exclaimed that she was blind. She was helped into a chair, and, in less than ten minutes (perhaps five) was dead. There was no struggling; the countenance remained quite placid. She had been heard to complain of palpitation of her heart for two or three days before her death.

*Dissection.*—The *brain* was remarkably healthy; so were the *lungs* upon the whole, except that they were much congested. *Heart* rather large; much fat upon its surface and among its fibres; the valves healthy; all the cavities contained a great quantity of fluid blood like tar: upwards of three pints flowed from the section of the great vessels. There was a remarkable accumulation of fat, and also many enlarged bronchial glands, closely applied around the base of the heart. "The loose cellular tissue about the apex of the pericardium had become converted into a dense steatomatous tumour, which had insinuated itself between the vessels, and left visible marks, in the alteration of form which had followed, of permanent compression of the pulmonary arteries and veins." The parenchymatous viscera of the *abdomen* were much congested.

Here then was an example of genuine Asphyxia.

The next *case* (No. 5) is in many respects similar to the preceding one. An old woman was in the act of dressing herself, when she suddenly became faint, sank into a chair, and in a few minutes expired. She had been complaining, the day before, of dyspnœa.

*Dissection.*—In this case, too, there was an immense mass of enlarged and indurated bronchial glands—one or two of which were almost as large as a hen's egg—surrounding the lower part of the trachea and the bronchi, which were flattened and compressed. The great blood-vessels also must have been subjected to considerable pressure, when the morbid mass was turgid with blood. The *lungs* were saturated with fluid blood. The *heart* was rather large. The was head not examined.

*Case* 6.—An idiot boy, 10 years of age, was found dead in his bed. He had never been affected with epilepsy. On *dissection,* the chief morbid appearance found was the *thymus* gland of very great size, mottled with intense congestion. The *thyroid* also, and the communicating slip between the two lobes, were in a similar condition. When full of blood, they must have caused considerable pressure upon the great vessels and bronchi. The whole venous system was much congested and loaded with blood.

The enlargement of the Thymus in this case deserves notice, from the circumstance that this lesion is believed to have something to do with some alarming chest diseases in children.

Case 7 is entitled, *Emphysema of the Lungs, Fat Heart, Repletion, Exercise, Exposure to Cold; sudden Death within five minutes.* The most conspicuous necroscopic appearance was enormous distension of the right cavities of the *heart* with fluid blood: the walls of the ventricle on this side were much thickened. Both *lungs* were emphysematous; their central parts loaded with blood and serum. The *brain* was sound.

*Case* 8 was similar in most respects to the preceding: the patient died within five minutes after the attack.

*Case* 9 was one of sudden Hæmoptysis. A cachectic young man, while carrying a box of medicines up stairs, was seized with a copious exspuation of blood. He died within five minutes of the attack. On dissection, the *lungs* were found to be deeply congested, and had acquired a spotted appearance in parts, from blood having been drawn into the air-cells. The *heart* was sound. The liver, spleen, and kidneys were immensely hypertrophied,* and soft and pale in texture.

---

* This term is greatly misused in the present day. The viscera mentioned were doubtless much enlarged in dimensions; but surely the enlargement was not from *over-nutrition.*

*Case* 10 affords another example of Asphyxia from pulmonary hæmorrhage. The patient, æt. 33, was phthisical. While straining and coughing upon the close-stool, he began to spit blood, and continued doing so until his death, in about 12 minutes from the moment of the attack.

*Dissection.*—In the upper lobe of the left lung was a large irregular vomica, from which the hæmorrhage appeared to have taken place; for along its wall ran a branch of the pulmonary artery, almost entirely denuded of surrounding tissue. Water thrown into the pulmonary artery escaped abundantly into this vomica, and apparently from the denuded vessel, where it was dipping into the pulmonary tissue; but the existence of an opening in it could not satisfactorily be ascertained. In several parts, the blood appeared to have been drawn into the areolar tissue of the lung, small patches of increased redness and density being scattered about. Heart healthy.

*Case* 11 is entitled *Hæmoptysis—Obstructive Disease of the Heart—Sudden Death in the Night.*—The patient was found dead in her bed. She seemed to be quite well the night before. On *dissection,* blood was found throughout the air-passages generally. The *lungs* were greatly congested. The mitral valve was much puckered, and the left auriculo-ventricular opening considerably contracted.

In the three preceding cases, death seems to have been occasioned by the obstruction to the admission of air into the pulmonary cells from the extravasation of blood. " The chief danger, under such circumstances, would seem to arise from the suddenness and extent of the hæmorrhage, the blood being poured out more rapidly than its evacuation can be effected; in addition to which, it coagulates about the fauces and mouth, especially when the expulsive efforts on the part of the patient begin to fail. When these conditions are most effectually in operation, asphyxia is accomplished as speedily as in cases of drowning, strangulation, and the like; as was evidenced in Case 9, where life became extinct in from three to four minutes from the moment of seizure."

### III. *Cases in which Death was the result of Syncope.*

Five examples are given under this head. In the first, the death was almost instantaneous from *rupture of the left auricle.* The pericardium contained nearly 20 ozs. of blood.

The next case is more interesting from the more gradual sinking of the patient, who was 59 years of age. Dr. Francis says:

" At 10 A.M. on September 22, 1844, I was sent for to visit him. He had been taking a short walk, and whilst in the act of practising upon the violincello, previous to playing at church, he was seized with intense pain across the forehead, faintness, and vomiting. He was sitting with his body bent forwards: the face was pale, shrunk, and expressive of agony: the pupils very slightly dilated. The intellect was unimpaired: the heart's action was very faintly perceived; the pulse scarcely at all.

" He was conveyed to bed; but died in about one hour and a half from the commencement of the seizure, having previously experienced some severe pains in the cardiac region." 89.

*Dissection.*—The pericardium contained about 26 ozs. of blood. *Heart* not much affected; but the aorta greatly diseased. Its internal coats were very brittle, and lacerable. At one point there was a round ragged opening in the external coat, large enough to admit a crow-quill; and about half an inch lower down there was a longitudinal slit in the internal coat of the vessel. Thus the lacerations in the two tunics did not correspond. Hence the cause of the extravasation of blood into the pericardial sac having been slow and gradual. The circumstance of this patient having been suddenly seized with "intense pain across the forehead, faintness, and vomiting," might naturally have suggested the idea that it was a cerebral attack. Perhaps it was so in part; for, although the substance of the brain appeared on dissection to be quite sound, its arteries were extensively ossified, so as in some parts to be nearly obliterated.

In *Case* 16, the sudden death was owing to the *rupture of an Aneurism* of the descending thoracic aorta into the left pleura. The heart was sound. The other two cases under this head need scarcely be mentioned. In one, there was a large aortic aneurism: the patient died almost immediately after attempting his own life, although he inflicted scarcely any injury upon himself.

### IV. *Cases wherein Death resulted from Epilepsy.*

Three examples are given. In two, the sudden death occurred during the night in bed. In the third, the patient had had several fits during the day of her decease: after one, at 9 P.M. being at the time in bed, deep coma supervened, and she died in a few minutes after its commencement. In none of these cases, did dissection reveal any thing conclusive as to the cause of death. How unsatisfactory upon the whole is the necroscopic history of this disease, Epilepsy!

Here we close our notice of this valuable paper with the following interesting extract. "In the absence of previous history, and under circumstances which afford but little time for deliberate reflection, the observation of the condition of the pulse and breathing would seem, so far as the few cases herein cited will allow, to be available with readiness in the diagnosis of the causes of these sudden deaths: at the least, encouragement is given for further investigation. It has fallen within the experience of most persons to have met with such cases, where, in the absence of post-mortem examination, various interpretations have been put upon the symptoms, whether referrible to lesion in the head or chest, by different observers who have been present. Thus the sudden approach and ingravescence of the attack are not at all times in favour of the heart, as is borne out by the three cerebral cases, especially Case 3. A pallid or livid face may be compatible with most of the varieties of sudden death; and in those cases wherein the state of the pupils is noticed, no uniformity was observable. Again, intense pain in the head, which, if present, might seem fairly to indicate a cerebral lesion, was most strikingly evidenced in the case of I—— (Case 13), who died from hæmorrhage into the pericardium. Vomiting also was a prominent symptom at the advent of this man's seizure. In fact, most of these signs are indicative merely of certain accidental complications not necessarily connected with the fatal lesion; and may therefore exist with lesions of totally different characters, provided only that the conditions upon which they depend happen to be present."

Our readers will probably have observed, from the preceding cases, how frequently the fatal attack appeared to be owing to the straining that attends the act of defæcation.

### IV. Reports of Cases of Diseases of Children, with Remarks. By *Golding Bird*, A.M. and M.D.

The first subject discussed and illustrated with cases is *Remittent Fever*. Sixteen cases were admitted into the children's ward during the twelvemonth, 1843-4, and about seventy were treated as out-patients.

"Under the general term," says our author, "of infantile remittent, gastric, or worm fever, most authors have, I think, confounded two very distinct affections; one depending almost entirely on derangement of the functions of the gastro-intestinal tube; the other upon more or less distinct malarious influence. A child previously well, partakes of unwholesome or indigestible food, and soon becomes irritable, peevish, and morose; loses its appetite; picks its face and nose; has an unpleasant odour of the breath, with a more or less tumid belly, some sickness and irregularity of bowels. Fever comes on, generally having pretty regular exacerbations; the tongue becomes furred; and a short, hacking cough appears. This state of things may go on for almost any period; under judicious treatment, and even without treatment in robust children, ending, in the great majority of cases, in health; whilst in strumous children the powers may become so impaired, that mesenteric disease, or obscure brain affection, may be ultimately induced, which may, and often does, prove fatal. The subject of such symptoms is said to be labouring under gastric or worm fever, whether accompanied by the development of ascarides or not. It is true that remissions, often distinctly marked, occur; but still there are, I think, sufficient grounds of distinction to allow us to diagnosticate this ailment from the *true remittent fever*, which, so far as I have seen, is never met with, save under circumstances involving more or less malarious influence. The true remitting fever, of which the first three cases may be taken as examples, prevails most generally during spring and autumn. The autumnal epidemic having, during the last two years, been, what I believe it generally is, the most severe. In both years it occurred most abundantly in September, and generally nearly disappeared in January. Almost all the cases occurring under treatment at the hospital were in children of families residing at Bermondsey, Rotherhithe, Deptford, and other places sufficiently near the banks of mud left by the Thames at each ebb-tide, to explain the possibility of the malarious origin of the disease. In the epidemic of the autumn of 1843 the disease was peculiarly severe in the districts alluded to, and existed contemporaneously with ague, which was particularly frequent among the applicants for relief at the hospital." 115.

The two sets of cases are, or at least ought to be, easily discriminated. In the *gastric* remittent fever, the disease is chronic; the alvine evacuations are always disordered and offensive; and the febrile paroxysms are never so severe, nor the remissions so complete, as in the *malarious* form of the disease. The latter generally attacks suddenly, often in the midst of robust health, and may frequently be as promptly subdued by appropriate treatment. Occasionally, indeed, both forms of the fever are co-existent; the one being superadded upon the back, so to speak, of the other. Such cases require, as a matter of course, more tact in their management.

Now for the *treatment;* and first of that of *Gastric Remittent Fever.*

"The use of mild mercurials, especially of the valuable pulvis sodæ compositus* of Guy's Pharmacopœia, in doses of three to eight grains at night, and a full dose of the pulvis rhæi salinus,† given every morning for a week or longer, was in almost every case successful. The latter compound is well known to the profession for its peculiar and almost unique influence in these affections; so much so as, in my mind, almost to justify the elaborate praise accorded to it by Dr. Fordyce seventy years ago.‡" 119.

Two or three grains of the Hydrargyrum cum creta—to which a grain of Ipecacuan is added—at bed-time, and a dose of infusum Sennæ, Manna, and carbonate of Soda in the morning, will be found excellent substitutes for these medicines, in certain cases. Rigid attention to the *diet* of the child is an indispensable item in the treatment. Farinaceous substances, milk, and light broths are the best articles of food.

*Treatment of Malarious Remittent Fever.*—The following plan was adopted by Dr. Bird.

"As a general rule, the hair has been cut short or removed; the surface of the body daily washed, with or without the use of the warm-bath; the diet extremely spare; and for medicine, after clearing out the bowels by hydrarg. c̄ cretâ, given in a single dose, or repeated each night, followed by a moderate dose of ol. ricini, the sodæ sesquicarb. in small doses has been generally preferred. As soon as the state of the secretions has been corrected, and the tongue become freer from fur, the paroxysmal exacerbations being distinctly marked, the Quinine has always been given. In a short time the remissions become longer and more complete, and in a space of time, seldom exceeding a week, the child has been convalescent. I have often been astonished to observe how almost specifically the quinine has acted in some of the severest cases. In one instance, when I had occasion to be at the hospital late at night, I found the little boy, William J—— (Case 1) with an ardently hot skin, with face almost scarlet, a sharp pulse, almost too rapid to be counted, and delirium; in fact every symptom of fever so intense, as, in the minds of some of the pupils who were with me, to justify most active antiphlogistic treatment; and yet the next morning, a remission having occurred, he bore the quinine in two-grain doses, and within five days the child was convalescent." 116.

When the remissions were not very distinctly marked, an emetic generally served to produce this effect; and then the Quinine was given, and it invariably cured the disease.

In many of the cases, an eruption of Urticaria, and sometimes of Erythema, occurred during the hot fit of the fever.

---

* "Sodæ Carbonatis exsiccatæ ʒv. Hydrargyri Chloridi ʒi. Pulv. Cretæ compositi ʒx. m."

† "Rhæi radicis pulv. ʒi. Potassæ Sulphatis ʒij. m."

‡ "Had I been more ambitious of dying a rich man, than of living an useful member of society, the powers of our anti-hectic powder in curing, as if by miracle, the hectic fever and the swelled bellies of children in this town would have remained a secret while I lived.—*Fordyce on Fever, Third Edition,* 1777, *p.* 228."

*Acute Croup.*—One severe case of this disease, successfully treated, is related. Warm baths, leeches to the throat, the use of emetics, and of frequently-repeated doses of calomel and tartrate of antimony, were the remedies employed. The temperature of the ward was raised to 68°, by an additional stove placed near the child's bed; and a dish of water was placed on the hot plate, so as to keep the air of the ward constantly moist. Dr. Bird thus comments upon the treatment that was pursued.

"That the loss of blood by leeches was of great service no one can doubt; although, probably, it would have succeeded better had it been earlier had recourse to. The antimony must, however, be, I think, regarded as the great remedy in this case. The proper action of the skin, maintained for so long a time, and kept up by moist hot air surrounding the patient, must have exerted an effective derivative influence from the inflamed mucous membrane of the larynx and trachea. The good effect of the hot moist air was not limited to the skin; for, entering the air-passages at each inspiration, it must have acted as a local vapour-bath to the inflamed tissues, and have certainly, to some extent, relieved the dyspnœa of the little patient. From what I witnessed in this case, and in two others since, in private practice, I feel inclined to urge upon the practitioner the propriety of keeping the chamber of the patient with croup as near 80° as possible, by means of closed doors and a good fire, and loading the atmosphere of the room with aqueous vapour, by keeping water constantly evaporating in some open vessel. In this case opium was early given in the form of compound tincture of camphor; believing it to be of great service in warding off spasm of the glottis, which certainly is in very many instances the immediate cause of death." 123.

*Infantile Syphilis.*—Several cases of syphilitic eruptions in young children are recorded. They were all promptly cured by small doses of Hydrargyrum cum Cretâ, given at bed-time, and a grain or so of Iodide of Potassium in syrup or decoction of Sarsa two or three times a day. The little creatures generally seemed to fatten on these medicines, so well did the treatment appear to suit. The diagnosis of infantile syphilis is usually abundantly obvious.

"The characteristic *snuffling* will often enable the practitioner to recognise the existence of disease, even before he has confirmed his opinion by visual examination. The puckered mouth, the position of the very characteristic eruption round the lips and anus, in addition to the peculiar varnished and fissured appearance of the parts from which the scales have faded, will seldom if ever fail to convert a suspicion of the true nature of the disease into positive certainty." 130.

*Dropsy after Scarlatina.*—This is a very frequent sequela of the fever in certain epidemics. In most cases, it seems to be traceable to a deficiency of the cutaneous perspiration, for a due length of time after the eruption has disappeared. Hence neglect of proper clothing and exposure to cold are the common exciting causes. The non-performance of the cutaneous functions induces a congestion of the kidneys, as shewn by the deficient quantity, deep colour, and the albuminous character of the urine;* and, as

* Besides albumen, the Urine in scarlatina often contains red particles of the system is not duly relieved by this secretion, the consequence is, that an effusion of serum takes place into the subcutaneous cellular tissue, and sometimes also into some of the internal cavities, of the body. It may seem strange, but it is not the less true, that Anasarca is more frequent in those cases where the eruption has not been vividly out, than where it has. This may, perhaps, be owing to the circumstance that the child is more apt to be neglected and sooner exposed to the weather, when the rash has been indistinct.

Keeping these remarks in view, the therapeutic indication will at once be obvious. Warm baths, confinement to bed, the use of diaphoretics, and the application (in some cases) of a large bran or linseed poultice to the loins, constitute the most simple and, generally too, the most successful line of treatment. If we have reason to expect great congestion of the kidneys, it will be prudent to draw a little blood by cupping from the renal region. The anasarca is usually observed to decrease *pari passu* with the appearance of sensible perspiration upon the surface, and the disappearance of the albumen from the urine. As the patients are often left weak and anæmiated after such attacks, the exhibition of some mild chalybeate will be found of much use.

Besides dropsical effusions, there is a marked tendency to the development of insidious inflammation of the serous membranes—more especially of the arachnoid, pleura and pericardium—in certain constitutions after an attack of scarlatina. Some patients again suffer from spasmodic pains in the limbs, but without there being any tumefaction or redness of the joints—a bastard sort of rheumatism. These pains generally yield to the use of warm baths and antimonials. We need scarcely say that the state of the urine should always be carefully examined in this as well as in the other sequelæ of scarlet fever. As it is highly probable that the train of effects—often of a grave character—following this disease are, at least in a great measure, referable to the retention of the nitrogenised elements of the urine in the circulation, the following " simple and easy process for the detection of urea in the blood and serous fluids"—recommended by Dr. Bird—may deserve to be generally known.

" Allow the blood to coagulate, decant the serum, and agitate it violently with its own bulk of rectified spirit: a dense deposit of albumen occurs, and the mixture may be set aside for subsequent examination, or, if time permits, this may be proceeded with immediately. For this purpose, throw the whole on a filter, and evaporate the filtered fluid slowly to a drachm or two; then add to it an equal bulk of dilute nitric acid of the Pharmacopœia, and once more filter. The filtered fluid, collected in a watch-glass, may be slowly evaporated to a few drops, and, on cooling, feathers of nitrate of urea will form in the liquor. Should the crystallization be imperfect, the deposited nitrate may be re-dissolved in a few drops of water, the solution decanted, and once more slowly evaporated. By this simple process, requiring no apparatus beyond an evaporating dish, any one may satisfy himself of the existence of urea in serous fluids containing it. With ordinary care the evaporation may be performed on the hob of a parlour fire-place,

---

*blood*, and also a number of large organic globules. On the other hand, the Blood contains some of the elements of the *urine;* as proved by the existence of urea in it, as well as in the secretions derived from it.

especially if a piece of card-board is interposed between the evaporating dish and surface of the hob, to prevent any accidental elevation of the temperature to too high a point." 139.

Dr. Bird's paper closes with a few remarks

*On the Use of Alum in Pertussis.*

As a matter of course, it is only in the non-inflammatory stages of the disease that this, or any analogous, medicine should be exhibited. "I have not yet met with," says our author, "any other remedy which has acted so satisfactorily, or given such marked and often rapid relief to the child. The dose, in my hands, has generally ranged from two to six grains in children from one to ten years of age, repeated every four or six hours. For a child of two or three years the following formula has generally been employed:

Aluminis gr. xxv. Extracti Conii gr. xij. Syrupi Rhædos ʒij. Aquæ Anethi ℥iij. m. capiat coch. i. med. 6tâ quâque horâ.

"I have never met with any inconvenient astringent effects on the bowels during the exhibition of this remedy: on the contrary, in more than one instance, it produced some diarrhœa. The only obvious effects resulting from its use were, diminished secretion of a less viscid mucus, with marked diminution in the frequency and severity of the spasmodic paroxysms."

The sulphate of Zinc has similar virtues, and will often be found a valuable remedy, combined with the extract of Conium, in chronic Pertussis. The muriate of Ammonia has been highly praised by some practitioners. We can confidently recommend the Liquor Potassæ in any bitter tea: the infusion of Calumba is a good vehicle.

The article on *Injuries of the Chest* does not admit of notice, as it con-consists entirely of cases without any accompanying remarks.

Our readers will agree with us, we doubt not, in regarding the present Number of these Reports as a very good one. *Vale et perge* we would say to the talented editors.

---

